# The intervention effect of Aitongxiao prescription on primary liver cancer rats was evaluated based on high-throughput miRNA sequencing and bioinformatics analysis

**DOI:** 10.3389/fonc.2023.1050069

**Published:** 2023-05-29

**Authors:** Lijing Xu, Jinlai Cheng, Zhuoxian Li, Xiaoyu Wen, Yuhao Sun, Meng Xia, Jing Leng

**Affiliations:** ^1^ Basic Medical College, Guangxi University of Chinese Medicine, Nanning, China; ^2^ Institute of Chinese Materia Medica, China Academy of Chinese Medical Sciences, Beijing, China; ^3^ Rehabilitation College, Guilin Life and Health Career Technical College, Guilin, China; ^4^ Institute of Microbiology and Genetics, Department of Molecular Genetics, University of Göttingen, Göttingen, Germany; ^5^ Guangxi Key Laboratory of Translational Medicine for Treating High-Incidence Infectious Diseases with Integrative Medicine, Guangxi University of Chinese Medicine, Nanning, China

**Keywords:** Aitongxiao prescription, primary liver cancer, exosomal microRNAs, high-throughput sequencing, bioinformatics analysis

## Abstract

Liver cancer is a common malignant tumor known for its difficult treatment and poor prognosis. As a traditional Chinese medicine prescription, Aitongxiao prescription (ATXP) has been used in clinical treatment of primary liver cancer (PLC) for more than ten years, and its therapeutic effect is obvious and has been verified over time. However, the mechanism of ATXP in treating PLC has not been fully elucidated. This study aimed to detect the liver-protective effect of ATXP on a PLC rat model and explore its potential mechanism from the perspective of plasma extracellular vesicle miRNAs. Fifty SPF male SD rats were randomly selected, with six rats as the control group, and the remaining rats were injected with DEN to establish a primary liver cancer model. The model rats were randomly divided into the model group and the ATXP group. After 4 weeks of intervention, the liver-protective effect of ATXP was evaluated using plasma biochemical indicators and histopathological methods. Plasma extracellular vesicles were isolated and extracted, and identified by transmission electron microscopy, nanoparticle tracking analysis, and western blot. Significant differentially expressed miRNAs in extracellular vesicles were screened by Illumina sequencing to explore the therapeutic targets of ATXP and conduct functional analysis. The results showed that ATXP significantly reduced plasma liver function in PLC rats and alleviated liver pathological damage. In addition, plasma extracellular vesicles were isolated and identified. According to the results of GO and KEGG analysis, they were related to multiple biological processes and covered multiple signaling pathways (PI3K-Akt and MAPK signaling pathways, etc.). The interaction between miR-199a-3p and MAP3K4 was determined by bioinformatics methods and dual-luciferase reporter gene detection, confirming that MAP3K4 is the target gene of miR-199a-3p. In conclusion, ATXP protects the liver from DEN-induced PLC, which may be related to the regulation of plasma extracellular vesicle miR-199a-3p. This study further reveals the mechanism of ATXP in treating liver cancer and provides a theoretical basis for subsequent research.

## Graphical abstract

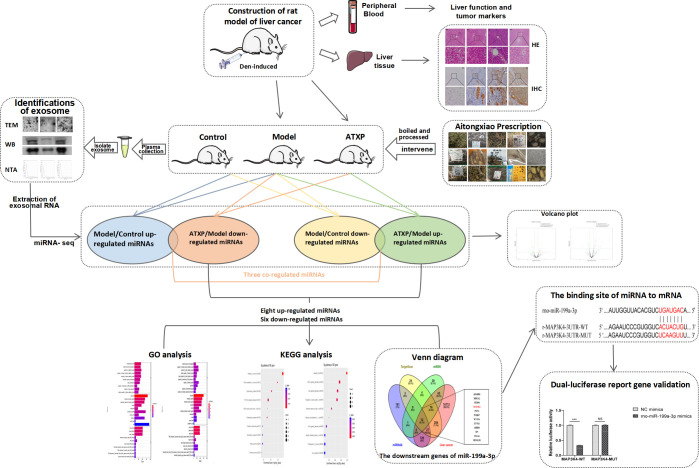

## 1 Introduction

Primary liver cancer is the most common fatal malignant tumor worldwide and is characterized by easy recurrence after surgery, short survival, and poor prognosis (1). According to the report on cancer incidence and mortality in the Global Cancer Case Study in 2018, liver cancer deaths accounted for 8.2% of global cancer deaths, ranking third (2). At present, the incidence and mortality of liver cancer in China are higher than those in other countries, and patients with liver cancer account for more than half of the total cases (3). Due to the insidious onset and rapid progression of liver cancer, the majority of patients are not diagnosed until the middle or late stages, missing the optimal time for surgical treatment. As patients have poor immune function and cannot tolerate radiation or chemotherapy, there is a lack of effective treatment options, resulting in poor treatment outcomes and prognosis. This often leads to serious health and economic burdens for patients (4).

Traditional Chinese medicine (TCM) is a unique original medical system in our country, has the characteristics of holistic treatment, and is widely used in the clinical diagnosis and treatment of liver cancer, which can effectively alleviate the clinical symptoms at different stages in patients with liver cancer, reduce the side effects of radiation and chemotherapy, and regulate the body’s immunity, thus helping to improve the quality of life and prolong the life span of cancer patients (5). Therefore, lessening the side effects and improving tolerance to traditional Chinese medicine that will benefit more patients are particularly important. Aitongxiao prescription was developed by Professor Ai-ling Wei of the First Affiliated Hospital of Guangxi University of Chinese Medicine. Compared with the original prescription, this prescription contains a greater variety of herbs aimed at fortifying the vital energy, clearing heat and toxins, and facilitating the circulation of qi and blood. Its primary function is to disperse blood stasis and toxins, while also boosting the vital energy and fortifying the body.

Traditional biomarkers for the prognosis, diagnosis, and monitoring of liver cancer, such as alpha-fetoprotein, have limitations not only in terms of the underlying mechanisms at the surveillance stage, but also in predicting late clinical outcomes. To address this issue, researchers have discovered extracellular vesicles with lipid bilayer membranes, which contain short non-coding RNAs called miRNAs that are widely involved in individual growth and development, particularly in the development (6), invasion and metastasis (7), drug resistance (8), immune regulation (9) and other aspects of tumors.

Building upon the aforementioned, this project aims to utilize the diethylnitrosamine (DEN)-induced primary liver cancer rat model to ascertain the efficacy of the model and evaluate the therapeutic potential of ATX prescription for primary liver cancer rats (10). This will be achieved by analyzing the four liver function indexes in plasma, the expression levels of AFP and GST-Pi, and the pathological status of liver tissue. To gain a comprehensive understanding of the mechanism of traditional Chinese medicine at the network level and omics perspective, we meticulously extracted plasma exosomes from each group of rats and conducted a thorough analysis of the miRNA in the exosomes. By doing so, we aimed to identify potential targets and shed light on the intricate mechanism of action of traditional Chinese medicine. Our ultimate goal is to develop novel methods and strategies for treating PLC.

## 2 Materials and methods

### 2.1 Preparation of ATXP

ATXP is composed of 17 herbs including *Mollugo stricta* Linn., *Spreading Hedyotis Herba*, *Rhizoma Curcumae*, *Lobeliae chinensis Herba*, *Trionycis Carapax*, *Chinemys reevesii*, *Ostrea gigas* Thunberg, *Aurantii Fructus*, *Radix Astragali*, *Codonopsis pilosula*, *Eucommiae Cortex*, *Sparganii Rhizoma*, *Persicae Semen*, *Radix Paeoniae Rubra*, and *Radix Glycyrrhizae Preparata* (**Table 1**). The herbs were purchased from the Pharmacy Department of the First Affiliated Hospital of Guangxi University of Traditional Chinese Medicine. They were authenticated by the Department of Teaching and Research of Chinese Medicine. The raw herbs were analyzed using high-performance liquid chromatography-mass spectrometry to determine the quality of ATXP as previously described (11). Before preparing ATXP, we soak it in a suitable amount of water for 30 minutes. First, add pure water 10 times the amount of herbs and heat it up for two times (boiling for 1 h each time). Then, use a vacuum pump filter, combine two volumes of the filtrate and divide it into separate containers. Finally, concentrate the filtrate and sterilize it, and store it at -20°C for later use. The concentration of the ATX prescription is approximately 2.57 g/ml.

**Table 1:** Composition of the Aitongxiao prescription (ATXP).

Latin name	Herb name	Chinese name	Family	Medicinal parts	Dosage (g)
Mollugo stricto Linn.	Carpetweed	Jie Du Cao	Apricotaceae	Herba	20
Spreading Hedyotis Herba	Hedyotis diffusa	Bai Hua She She Cao	Rubiaceae	Herba	15
Rhizoma Curcumae	Curcuma zedoaria	E Zhu	Ginger	Rhizoma	10
Lobeliae chinensis Herba	Lobelia chinensis	Ban Bian Lian	Campanulaceae	Herba	15
Triorrycis Carapax	Turtle nail	Bie Jia	Turtle	Shell	10
Chinemys reevesii	Tortoise shell	Gui Jia	Turtle	Shell	10
Ostrea gigas Thunberg	Oyster	Duan Mu Li	Oyster	Shell	10
Aurantii Fructus	Fructus aurantii	Zhi Qiao	Rutaceae	Fruit	10
Radix Astragali	Astragalus mongholicus	Huang Qi	Papilionaceae	Root	15
Codonopsis Pilosula	Codonopis	Dang Shen	Campanulaceae	Root	10
Eucommiae Cortex	Eucommia ulmoides	Du Zhong	Eucommia	Bark	10
Sparganii Rhizoma	Common burreed rhizome	San Leng	Sparganiaceae Hanin	Rhizoma	10
Persicae Semen	Peach kernel	Tao Ren	Rosaceae	Seed	10
Radix Paeoniae Rubra	Red peony root	Chi Shao	Ranunculaceae	Root	15
Radix Glycyrrhizae Preparata	Glycyrrhiza uralensis Fisch.	Zhi Gan Cao	Legurninosae	Root and Rhizomes	5

### 2.2 Model preparation and grouping intervention of rats

A total of 50 healthy SPF-grade male Sprague–Dawley (SD) rats, 6–8 weeks of age, weighing 180–200 g, were purchased from Tian Qin Biological Technology Co., Ltd. (Changsha, China). The production license number of these rats is SCXK (Hunan) 2014-0011.

The animals were placed in an SPF standard room of Guangxi University of Traditional Chinese Medicine with good ventilation. The light/dark cycle lasted for 12 h and the appropriate temperature was 22°C. All experimental procedures were carried out in accordance with the Guidelines for Experimental Animal Health of the National Institutes of Health and approved by the Animal Ethics Committee of Guangxi University of Chinese Medicine (Ethical approval code: DW20201226-91, Guangxi, China).

**Table 2** shows the whole process of animal experiments from model preparation to TCM intervention. After 1 week of adaptive feeding, six rats were randomly taken as the control group, and the remaining rats received intraperitoneal injections of diethylnitrosamine (70 mg kg^−1^ week^−1^). The modeling process lasted for 10 weeks and stopped at weeks 11 to 16. The rats were weighed and their weights were recorded once a week. The general conditions such as the activity and spirit of the rats were observed, and the death of each group was recorded. Three rats in the experimental group were killed at 0, 6, l3, and l6 weeks after cancer induction, and the liver tissue was taken, sectioned, and stained to observe the carcinogenesis. At the beginning of 17 weeks, 25 rats modeled successfully were randomly classified as the model group (*n* = 12) and the treatment group (*n* = 13). The treatment group was administrated with ATX decoction (18 g/kg), which was the optimal concentration of the ATXP. The control group and the model group were given an identical amount of distilled water. The rats in each group had different interventions for 4 weeks.

**Table 2:** The animal experimental design from model preparation to TCM intervention.

Group	Week 1–10	Week 11–16	Grouping intervention	Week 17–20	Last day of week 20
Control (*n* = 6)	Intraperitoneal injection of normal saline	Stop the intraperitoneal injection of anything	Control (*n* = 6)	Distilled water	Euthanasia
Model preparation group (*n* = 44)	Intraperitoneal injection of diethylnitrosamine (70 mg·kg^−1^·week^−1^)		Model (*n* = 12)	Distilled water	
			Treatment group (*n* = 13)	18 g·kg^−1^·day^−1^ of ATXP	

### 2.3 Liver function assays and tumor marker evaluation

Rats were injected intraperitoneally with 3% sodium pentobarbital (45 mg/kg). After anesthesia, 10 ml of blood was taken from the abdominal aorta and then centrifuged for 10 min at 1,200 r/min. The plasma samples were isolated from the blood, and the supernatant was harvested and finally stored in a −80°C refrigerator for subsequent use. Aspartate aminotransferase (AST) and alanine aminotransferase (ALT) in rat plasma were detected by an automatic biochemical analyzer, gamma-glutamyl transpeptidase (GGT) and α-L-fucosidase (AFU) were tested by using a spectrophotometer, and alpha-fetoprotein (AFP) was detected by an ELISA kit (Bioswamp, Shanghai, China), respectively, following the manufacturer’s instructions.

### 2.4 Histomorphological observation of rat liver

Liver (tumor) tissues from the blank, model, and ATXP groups were excised and fixed with 4% paraformaldehyde (Sinopharm Chemical Reagent Co. Ltd., China) for 24 h. Fixed tissues were cut into 1.5 × 1.5 × 0.3 cm sections, dehydrated in a gradient alcohol series, and embedded in paraffin wax blocks. The tissue was cut into 4–5-μm-thick sections and mounted on glass slides. After dewaxing in xylene, the slides were dipped in hematoxylin and agitated for 3–6 min, rinsed in water for 1–2 min, and stained with 0.5% eosin Y solution for 2–3 min at room temperature. The slides were examined under a Leica DM1000 microscope (Leica, Germany). At least 3 visual fields were observed during the experiment, and each field was photographed at 100 μm scales. Finally, the most representative visual field was selected for display.

### 2.5 Protein expression of GST-Pi in rat liver tissue

The liver (tumor) tissue was fixed with 4% formaldehyde and embedded with conventional paraffin to make 4–5-μm paraffin continuous sections. The GST-Pi kit was purchased from Fuzhou Maixin Company (Fuzhou, China). The operation steps were taken according to the instructions of the kit, and the relevant parts of the liver tissue sections were collected. The image was analyzed and its IOD value was calculated. At least 3 visual fields were observed during the experiment, and each field was photographed at 100μm scales. Finally, the most representative visual field was selected for display.

### 2.6 Isolation and identification of plasma exosomes

After anesthesia, 10 ml of blood was taken from the abdominal aorta and centrifuged at 1,200 r/min for 10 min, plasma samples were separated from the blood, and the supernatant was collected. The exosomes from plasma were separated and extracted by ultracentrifugation. The specific method is as follows: 1) centrifuge the liquid sample at 300×*g* for 10 min and then remove the cells from the sample; 2) transfer the supernatant to a new EP tube and centrifuge at 2,000×*g* for 10 min; 3) collect the supernatant and centrifuge at 10,000×*g* for 30 min to eliminate the shed microvesicles (sMV, 200–1000 nm); 4) collect the supernatant and centrifuge at 100,000×*g* for 70 min; and 5) wash in PBS and use a 0.22-μm membrane filter (Merck Millipore Darmstadt, Germany) filtration; and 6) the obtained plasma exosomes were stored at −80°C for later use. Transmission electron microscopy (TEM) was used to observe the morphology of the plasma exosomes, and a Tecnai G2 Spirit 120KV (FEI Czech Co. Ltd., Hillsboro, USA) was adopted to capture the images of the plasma exosomes. Nanoparticle tracking analysis (NTA) was utilized to determine the particle size and concentration using ZetaView PMX-120 (Particle Metrix, Germany). The surface markers of the plasma exosomes were detected by Western blot analysis. The following antibodies were used: Alix (sc-53540, Santa Cruz, Northern California, USA) and CD63 (ab134045, Abcam, Cambridge, UK). According to the instruction of the Qubit™ Protein Assay Kit, the concentrations of the exosome protein in the model, control, and ATXP groups were 1,805, 1,715, and 2,240 μg/ml, respectively.

### 2.7 Extraction of exosomal RNA

The exosome samples were removed from the refrigerator at −80°C, transferred to a 1.5-ml centrifuge tube, and mixed with TRIzol reagent. Chloroform was added, the tube cap was tightly closed, and the RNA in the sample was extracted after 15 s of vigorous oscillation of the tube body and centrifuged at 4°C 12,000 ×g for 15 min. After centrifugation, the solution was divided into phases, the water phase was transferred to a new tube, isopropyl alcohol was added, and the RNA in it was mixed to precipitate. After mixing, the solution was centrifuged at 4°C 12,000×*g* for 10 min. The supernatant was removed, 75% ethanol was added to the sample, and the RNA precipitation was cleaned. After oscillation, the supernatant was removed by centrifugation at 4°C 7,500×*g* for 5 min. The RNA solution can be obtained by adding RNA-free water to dissolve precipitation.

### 2.8 Microarray analysis of plasma exosome miRNAs

RNA in the exosome was extracted and quantified by quality inspection to construct the library, which was transformed into single-stranded DNA. Illumina NextSeq 500 sequencer was used to conduct 51-cycle sequencing. After sequencing, the "Solexa CHASTITY" technique was used to filter the raw "reads" for obtaining "clean reads". Then, the "cutadapt" software was used to remove the "adapters" and retain the tags equal to or greater than 15nt to obtain "trimmed reads". All "trimmed reads" were quantified for known miRNAs and predicted for new miRNAs using the "miRDeep2" software.

### 2.9 Bioinformatics prediction and analysis of plasma exosome miRNA target genes

To improve the accuracy of miRNA gene target prediction, the results were obtained from TargetScan (12), miRDB (13), and miRWalk (14). For the target gene prediction of miRNA, the GO and KEGG databases were used to conduct functional and pathway enrichment analyses of the target genes. Abundant biological processes, molecular functions, cell components, and signaling pathways were classified, and a miRNA target gene–gene functional regulatory mechanism network was established.

### 2.10 Dual-luciferase reporter gene assay

To identify the binding sites between miR-199a-3p and MAP3K4, luciferase structures containing MAP3K4 were transfected with wild-type and mutant binding sites, respectively, and co-transfected with miR-199a-3p mimics or empty vectors. To assess the luciferase activities of MAP3K4, cells were transfected with MAP3K4 luciferase constructs containing wild-type or mutated binding sites, cells were collected 48 h later, and luciferase activity was tested using a dual-luciferase reporting kit according to the manufacturer’s instructions.

### 2.11 Data analysis

SPSS 20.0, GraphPad Prism 8.0.2 and Adobe Illustrator 2022 were used for statistical analysis and mapping. All data were presented as mean ± standard deviation, using ( $\bar{x}$ ± *s*) as denotations. One-way ANOVA was used for the comparison between groups. The level of test significance at *α* = 0.05 was *p <*0.05. Differentially expressed miRNAs between two groups were performed according to the screening criteria of >1.5- or <0.67-fold change in expression and *p <*0.05.

## 3 Results

### 3.1 Establishment of a rat model of experimental primary hepatic carcinoma

**Figure 1:**
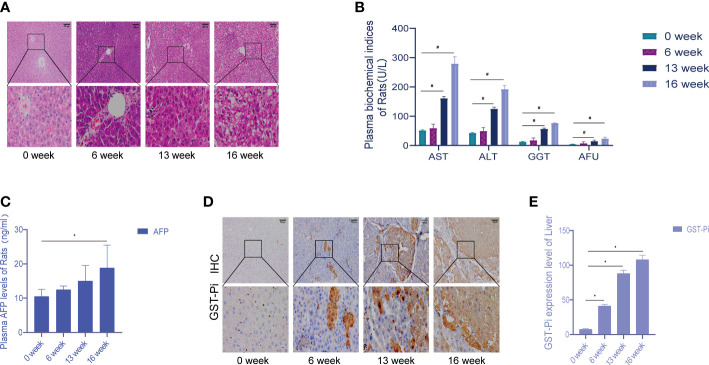
Establishment of the experimental primary liver cancer rat model. **(A)** HE staining of rat liver tissue (the pathological changes of the liver during 0–16 weeks). All images are shown at identical magnification; scale bar = 100 μm. **(B)** Changes in the contents of various biochemical indexes in rat plasma at different stages of modeling. **(C)** The changes in plasma AFP content in rats during 0–16 weeks. **(D)** Different stages of the immunohistochemical test. All images are shown at identical magnification; scale bar = 100 μm. **(E)** Differences in GST-Pi expression in rat liver tissues at different stages of modeling. *n* = 3 rats per group (*p < 0.05, ^#^ *p* < 0.01 compared with the 0 week; 0 week was the model preparation group before DEN administration).

Death cases began to occur from the 7th week of modeling, and a total of 7 rats died by the end of the 16th week. An autopsy found that a large amount of fluid remained in the abdominal cavity as well as some intestinal lesions, including one rat with lung metastasis. In the 6th week of modeling, the liver cells around the central vein swelled, showing vacuolar degeneration, and inflammation in the portal area was obvious; the liver lobule structure was still intact, and local necrosis and inflammatory cells were seen in the lobule infiltration. In the 13th week of modeling, the central vein hepatocyte edema and necrosis continued to increase, showing the presence of different degrees of steatosis; eosinophilic or clear cell hyperplasia foci appeared, the fibrous septum was more obvious, and typical pseudo-lobular formation was obvious. In the 16th week of modeling, the liver cancer cells were in masses, and the liver lobules were structurally disordered, accompanied by a large number of inflammatory cell infiltrations, some bleeding and necrosis, and hepatocyte hyperplasia and nodules. During the formation of rat liver cancer induced by diethylnitrosamine, the morphology and pathological sections of rat liver were changed correspondingly, which was consistent with the progression of DEN modeling from hepatitis to cirrhosis and then to liver cancer (**Figure 1A**). Compared with week 0, the plasma levels of ALT, AST, GGT, and AFU showed significant differences at 13 and 16 weeks of modeling (**Figure 1B**). There was no significant change in plasma AFP content at 6 and 13 weeks compared with week 0, but the difference in plasma AFP content at week 16 suggested that the liver tissue was damaged during DEN modeling (**Figure 1C**). **Figure 1D** showed the activity of GST-Pi in the liver (tumor) tissue. It could be seen that the GST-Pi protein expression in the liver of the model group gradually increased with the process of modeling. Compared with week 0, the expression of GST-Pi in the model group was significantly different at 6, 13, and 16 weeks (*p* < 0.05); there was no significant difference between the 13th week and the 16th week, but both showed an upward trend (**Figure 1E**).

### 3.2 The therapeutic effect of ATX prescription on the PLC rat model

**Figure 2:**
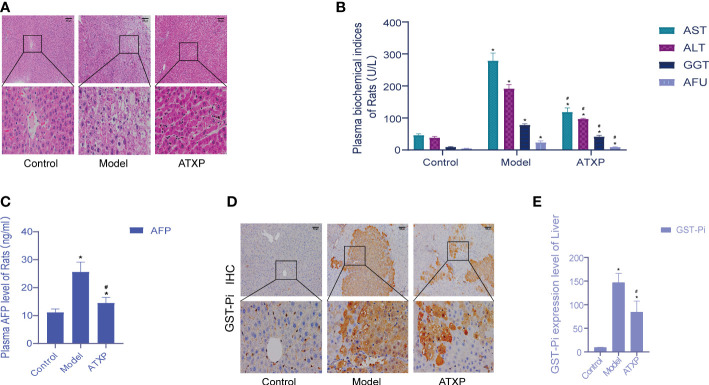
Examination of the experimental groups. **(A)** Liver histopathological examination of rats in each group after intervention. **(B)** Changes of plasma biochemical index content of rats in each experimental group. **(C)** AFP level of the experimental groups. **(D)** Immunohistochemical test. **(E)** GST-Pi expression in rat liver tissues of each experimental group. * compared with the control group, *p < 0.05; # compared with the model group, ^#^ *p* < 0.05 (control group: *n* = 6, model group: *n* = 12, ATXP group: *n* = 13).

The liver cells in the blank group were intact and radially arranged around the central hepatic lobule vein. Compared with the model group, liver biopsy showed that hepatocellular carcinoma cells in the model group were lumpy, and the liver lobule structure was disorganized, accompanied by a large number of inflammatory cell infiltration, hemorrhage, necrosis, and hyperplasia of the liver cells and nodules. The pathological sections of the liver of rats in the ATXP group showed reduced tumor cells and inflammatory cells, and a relatively orderly arrangement of liver cells was observed at high magnification compared with the model group. The liver morphology and pathological sections of rats in the ATXP group showed improved tumor and inflammatory cells, and a relatively orderly arrangement of liver cells was observed at high magnification (**Figure 2A**). The contents of ALT, AST, GGT, AFU, and AFP in the plasma of the ATXP group were significantly lower than those of the model group but higher than those of the control group (**Figures 2B, C**). As can be seen from **Figures 2D, E**, compared with the control group, GST-Pi protein in the liver tissue of the model group and the ATXP group was significantly higher, and that of the model group was significantly higher than that of the ATXP group. The results showed that GST-Pi decreased significantly after the ATXP intervention. In conclusion, ATXP has a regulatory effect on the DEN-induced rat liver cancer model.

### 3.3 Isolation and identification of rat plasma exosomes

**Figure 3:**
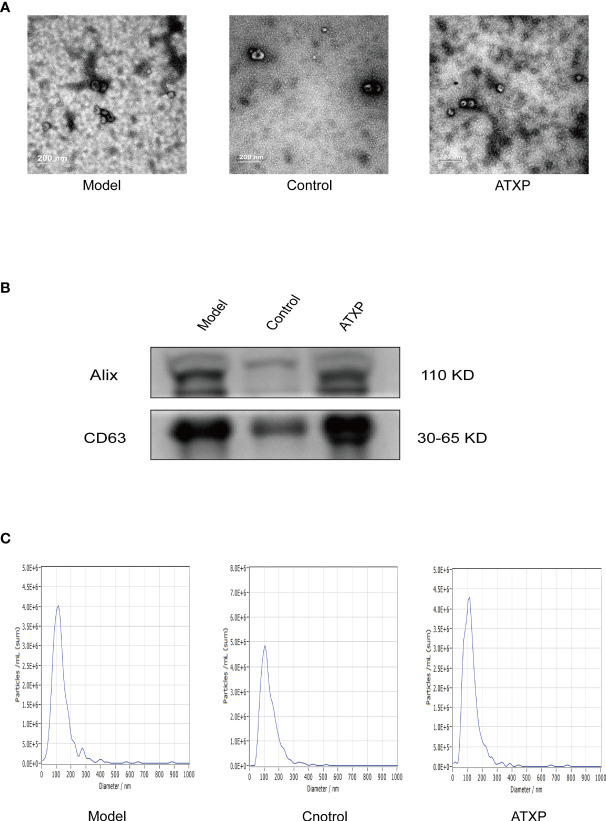
Characteristics of exosomes in the peripheral blood from the three groups. **(A)** Representative transmission electron micrograph images of exosomes derived from the three groups; scale bar = 200 nm. **(B)** Western blot analysis showed the presence of two common positive exosomal markers (Alix and CD63 in the exosomes isolated from the three groups). **(C)** Representative nanoparticle tracking analysis report of exosomes from the three groups.

The results of TEM showed that the extracted substance had a bilayer membrane structure and cup-shaped morphology, which was consistent with the morphology of exosomes (**Figure 3A**). WB results showed that exosomes in both groups expressed the exosomal marker proteins Alix and CD63. The results of NTA showed that the particle sizes of exosomes in the two groups were concentrated between 30 and 150 nm, which was consistent with the recognized exosome particle sizes (**Figure 3B**). The mean particle sizes of the model group, control group, and ATXP group were 109.2, 102.5, and 108.1 nm, respectively. The concentrations were 1.7 × 10^11^, 1.3 × 10^11^, and 8.6×10^10^ particles/ml (**Figure 3C**). According to previous studies, it could be identified as an exosome.

### 3.4 Quality of sequencing data

**Figure 4:**
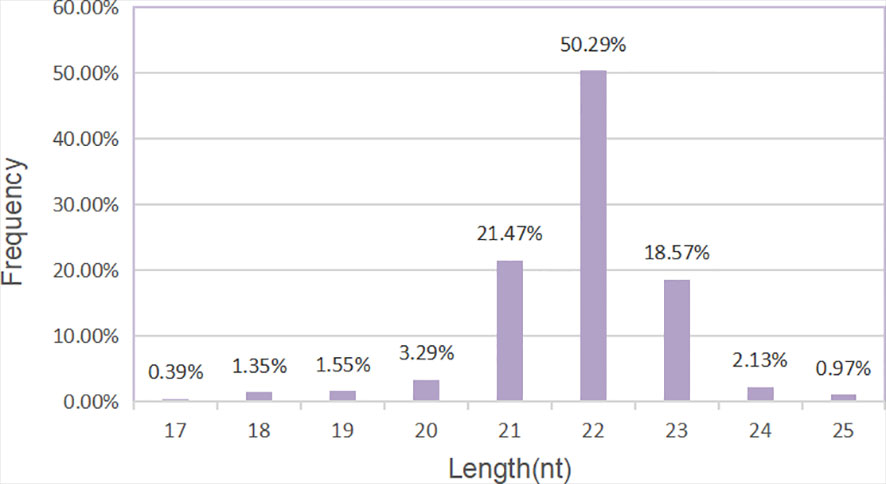
Length distribution of miRNAs. Histogram of length distribution: the abscissa is the length of miRNA, and the ordinate is the frequency of occurrence.

The sequencing data generated by Illumina NextSeq 500 are raw sequencing data. Quality control was carried out on the original sequencing data to evaluate whether the sequencing results were affected by quality differences in the process of library preparation. First, we analyzed the quality of sequencing data according to the distribution length of miRNAs. We could see that the label length of each group of samples was mainly distributed in 21–23 nt, which was consistent with the length distribution of miRNAs in conventional animal samples (**Figure 4**).

### 3.5 Differentially expressed profile of miRNAs

**Figure 5:**
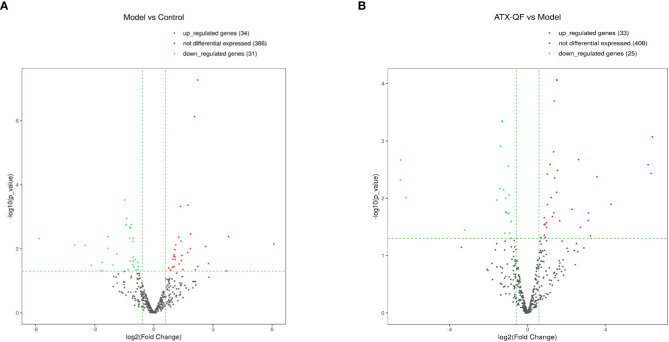
Three groups of significantly differently expressed miRNAs. **(A)** Volcano plot analysis of DEMs between the model group and the control group. **(B)** Volcano plot analysis of DEMs between the ATXP group and the model group. Note: The *x*-axis represents the log_2_ fold change value, and the *y*-axis represents the −log_10_ *p*-value. The two vertical green lines represent upregulation (right) and downregulation (left), and the green parallel lines correspond to the *p*-value threshold. Green represents significantly different downregulated miRNAs, red represents significantly different upregulated miRNAs, and gray dots represent non-significantly different miRNAs.

Differential analysis of miRNAs was performed according to the expression of miRNAs. The screening criteria for the difference was that the log_2_ FC ≥1 or ≤−1, and *p <*0.05. Based on the statistical results, we analyzed the differences in miRNA expression among the three groups. As shown in **Figure 5A**, for the model group compared with the control group, 34 miRNAs were upregulated, while 31 were downregulated (*p* < 0.05). We also analyzed the effect of Chinese medicine treatment on miRNA expression (comparisons between the ATXP group and the model group). It was found that 33 miRNAs showed expression changes in the ATXP group compared with the model group, while 25 were downregulated as shown in **Figure 5B** (*p* < 0.05). To investigate the effect of TCM treatment on the abnormal expression of exosomal miRNAs in the rat model of experimental liver cancer, we further analyzed the differences between the ATXP group and the model group, as well as the intersection of the differential molecules between the model group and the control group. A total of 3 miRNAs were found in the intersection of the differential molecules between the ATXP group and the model group and between the model group and the control group. As shown in **Table 3**, among the miRNAs differentially expressed between the model group and the control group, there were three reversals after the ATXP treatment, namely, miR-206-3p, miR-98-3p, and miR-431. miR-206-3p was significantly upregulated in the model group and was markedly downregulated after the ATXP treatment. miR-98-3p and miR-431were significantly downregulated in the model group and were markedly upregulated after the ATXP treatment. Subsequently, by analyzing the miRNA of the exosomes in two groups and exploring the effect of ATXP in the treatment of primary liver cancer, we conducted a comparative screening of tumor suppressor genes. The analysis results showed that there were 14 miRNAs with significant differences between the ATXP group and the model group (**Table 4**). Among them, the upregulated miRNAs in the ATXP group included miR-339-5p, miR-223-5p, miR-147, miR-223-3p, miR-431, miR-199a-3p, miR-98-3p, and miR-99a-5p. The downregulated miRNAs included miR-125b-1-3p, miR-134-5p, miR-206-3p, miR-9a-5p, miR-674-3p, and miR-133a-5p. These miRNAs were the target of treatment for PLC.

**Table 3:** Differential miRNAs in the model *vs*. control and the ATXP *vs*. the model group (log_2_ FC ≥1 or ≤−1, and *p* < 0.05).

Group	ATXP/model (downregulation) *vs*. model/control (upregulation)	ATXP/model (upregulation) *vs*. model/control (downregulation)
Co-regulated miRNA	rno-miR-206-3p	rno-miR-98-3p
		rno-miR-431

**Table 4:** Differential expression analysis of miRNAs in the ATXP and model groups (fold change >1.5 or <0.67, *p* < 0.05).

Group	Up/downregulation	Gene name	Fold change	*p*-value
ATXP *vs*. model	Upregulation	*rno-miR-431*	19.324	0.013
		*rno-miR-98-3p*	11.803	0.004
		*rno-miR-223-5p*	2.570	0.000
		*rno-miR-339-5p*	2.515	0.002
		*rno-miR-223-3p*	2.221	0.003
		*rno-miR-99a-5p*	2.016	0.004
		*rno-miR-147-5p*	1.806	0.044
		*rno-miR-199a-3p*	1.599	0.048
	Downregulation	*rno-miR-133a-5p*	0.336	0.011
		*rno-miR-9a-5p*	0.376	0.007
		*rno-miR-674-3p*	0.426	0.007
		*rno-miR-134-5p*	0.463	0.017
		*rno-miR-206-3p*	0.518	0.018
		*rno-miR-125b-1-3p*	0.531	0.041

### 3.6 Target miRNA regulation gene and function analysis

**Figure 6:**
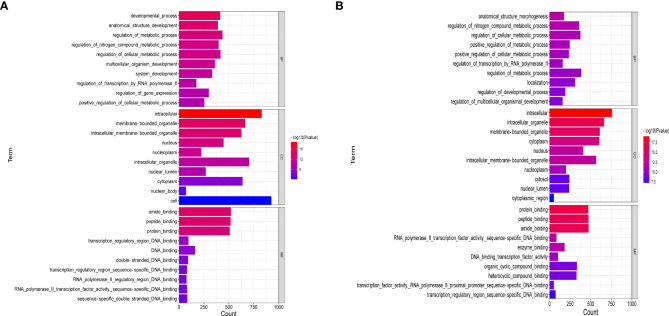
GO enrichment analysis of liver cancer targets treated by ATX prescription (BP, CC, MF). The 10 most enriched GO terms in biological process, cellular component, and molecular function for DEGs–miRNAs from the ATXP group *vs*. the model group are listed: **(A)** upregulation and **(B)** downregulation. Arranged according to *p*-values from low to high, and the ordinate represents the *p*-value (−log_10_ conversion).

**Figure 6** showed the enrichment analysis of differential miRNAs in order to identify the targets of these miRNAs differentially expressed between the ATXP group and the model group. GO analysis showed that the cell components of the eight miRNA targets were significantly upregulated in the intracellular, cytoplasm, intracellular membrane-bound, membrane-bound organelle, intracellular organelle, cytosol, etc. Their molecular functions were specifically expressed as protein binding, peptide binding, amide binding, enzyme binding, and transcription regulatory and participated in biological processes (e.g., cellular localization, regulation of the metabolic process, positive regulation of meta, protein localization, and cellular protein localization) (**Figure 6A**). **Figure 6B** showed that the cell components of the six miRNA targets were significantly downregulated in the cytoplasm, nucleus, nucleoplasm, cytosol, intracellular organelle, membrane-bound organelle, etc. Their molecular functions were specifically expressed as protein binding, peptide binding, amide binding, enzyme binding, and DNA binding transcription factor activity and participated in biological processes (e.g., anatomical structure morphogenesis, regulation of nitrogen compound metabolic, regulation of cellular metabolic, positive regulation of metabolic, regulation of transcription by RNA polymerase II, and organic cyclic compound binding).

### 3.7 KEGG analysis of target genes of differentially expressed miRNAs

**Figure 7:**
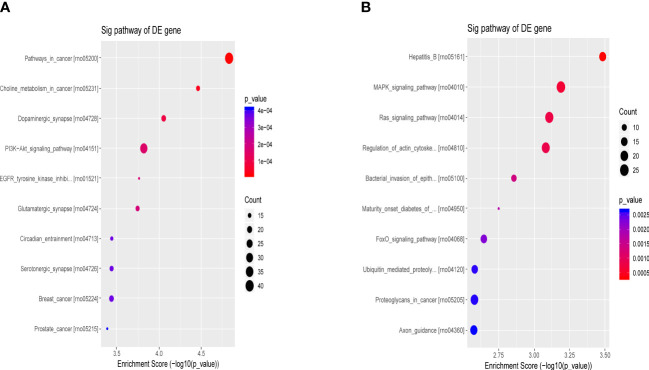
Bubble chart of the first 10 entries in the KEGG pathway by ATX prescription. The first 10 entries of KEGG pathways enriched for DEGs–miRNAs from the ATXP group *vs*. the model group: **(A)** upregulation and **(B)** downregulation. Arranged according to *p*-values from low to high, and the ordinate represents *p*-value (−log_10_ conversion). Different colors and diameters of the pathway dots represent significance level and gene number, respectively.

The differential molecular target gene function/signaling pathway between the ATXP group and the model group involved multiple pathways, e.g., the PI3K-Akt signaling pathway, MAPK signaling pathway, Ras signaling pathway, and FOXO signaling pathway, as illustrated in **Figure 7**. These signaling pathways were involved in cell proliferation, differentiation, apoptosis, autophagy, immune regulation, and other important biological functions (15–18).

### 3.8 Exosomal miR-199a-3p directly targets MAP3K4

**Figure 8:**
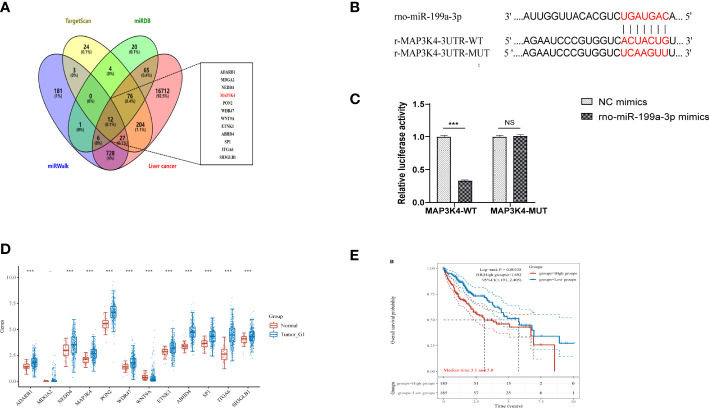
MAP3K4 is targeted and negatively regulated by miR-199a-3p. **(A)** Venn diagram of expressed prediction results of the downstream genes of miR-199a-3p, and the middle part represents the intersection target with liver cancer. **(B)** The binding site between miR-199a-3p and MAP3K4. **(C)** The binding of miR-199a-3p and MAP3K4 verified by the dual-luciferase report gene assay. **(D)** Expression of MAP3K4 in the liver tumor. **(E)** Survival analysis of MAP3K4 in different expression levels. "NS" means not statistically significant; *** P<0.001.

To determine the downstream regulatory mechanism of miR-199a-3p, we employed three bioinformatics tools (miRDB, miRWalk, and TargetScan) to predict the downstream genes of miR-199a-3p. Subsequently, we obtained liver cancer targets from the GeneCards database (https://www.genecards.org/), and finally selected the common targets among the four as candidate biomarkers (**Figure 8A**). According to the TargetScan database, miR-199a-3p and MAP3K4 exhibited potential binding sites (**Figure 8B**). A dual-luciferase reporter gene assay revealed that the luciferase activity of the MAP3K4-WT co-transfected with miR-199a-3p mimic was inhibited dramatically, while the luciferase activity of MAP3K4-MUT co-transfected with miR-199a-3p mimic did not change notably (**Figure 8C**). Subsequently, RNA-sequencing expression (level 3) profiles and the corresponding clinical information for liver cancer were downloaded from the TCGA dataset (https://portal.gdc.com). The gene expression of 12 candidate targets was compared. The results showed that MAP3K4 was highly expressed in hepatocellular carcinoma (HCC) (**Figure 8D**). Further prognostic analysis of MAP3K4 with different expression levels confirmed that highly expressed MAP3K4 had a poor prognosis in the past 5 years (**Figure 8E**). Therefore, we speculated that miR-199a-3p could target and negatively regulate MAP3K4, and the upregulation of MAP3K4 might eliminate the inhibitory effect of miR-199a-3p on liver cancer.

## 4 Discussion

In this study, the rat model of primary liver cancer was constructed by intraperitoneal injection of the chemical inducer DEN, which was convenient to operate and highly carcinogenic (19). Research showed that DEN had the dual effects of a medium agent and a cancer inducer, and the rat liver cancer model induced by DEN was similar to the human chronic disease process (20, 21). Through DEN modeling, it was found that rats would experience three distinct disease courses, namely, hepatitis stage (week 6), cirrhosis stage (week 13), and hepatocellular carcinoma stage (week 16), which were similar to human hepatocellular carcinoma in occurrence, development, and pathological changes, and were also ideal animal models for imaging and molecular biology studies. Based on this, we used the DEN-induced rat liver cancer model to study.

AST, ALT, AFU, and GGT are the frequently measured liver function indicators in clinical practice, which often indicate the degree of liver injury and have certain diagnostic value for the discovery of early liver cancer, while AFP is the most commonly used plasma marker for the diagnosis of liver cancer at present (22, 23). The levels of plasma ALT, AST, GGT, AFU, and AFP in the model group at 0, 6, 13, 16, and 20 weeks all gradually increased with the modeling time. Among them, the plasma levels of ALT, AST, GGT, and AFU had a significant difference from the blank control group in the 13th week of modeling, which was statistically significant (*p* < 0.01), and the AST/ALT ratio in each time period was greater than 1. Due to the high false-positive rate of single liver cancer detection index analysis, multiple liver diseases can lead to elevated AST and ALT values. AST/ALT >1 can be used as a marker to evaluate patients’ liver function and judge their prognosis, so the combination of AST/ALT >1 in the diagnosis can effectively exclude false positives caused by cholecystitis, gallstones, pancreatic tumor, and sclerosing cholangitis and improve diagnostic accuracy (24).

GST-Pi is a phase II metabolic enzyme and an important detoxification enzyme system for cell anti-damage and anti-carcinogenesis (25). As a pre-liver cancer indicator, GST-Pi is also abnormally expressed in a variety of tumors, and its expression level is directly proportional to the degree of malignancy and prognosis of tumors (26). GST-Pi is highly expressed in most precancerous lesions, and it is also highly abnormal in rat liver cancer lesions (27). According to the analysis of the GST-Pi protein expression in the liver tissues of the model group during each time period of modeling, it was found that the GST-Pi protein expression in the liver tissues of rats at 6, 13, 16, and 20 weeks increased gradually compared with the blank group and was statistically significant (*p* < 0.05). Therefore, the model was successfully established.

In the intervention experiment of ATXP on the model group, from the first week of TCM intervention, it had no significant effect on the overall situation of rats (including hair color, mood, food intake, and water intake). However, in the liver morphology and pathological changes of rats, it was found that ATXP had a positive regulatory effect on the liver cancer rat model and had a regulation effect on ALT, AST, GGT, AFU, AFP, and liver GST-Pi protein expression in a liver cancer rat model. Therefore, we believe that ATXP has an intervention effect on PLC rats.

In the gene-level regulation mechanism, miRNA has also become a research hotspot in recent years. miRNAs are short non-coding RNAs with the size of 18-23 nt. The first miRNA was discovered in *Caenorhabditis elegans*, and since then, a variety of miRNAs have been discovered in different animals and plants, which can regulate organism development, regulate the cell cycle, and participate in cell inflammation, proliferation, apoptosis, and other processes. Recently, many studies have shown that some miRNAs may inhibit tumor function, while other miRNAs may have carcinogenic effects (28, 29).

Exosomes are small vesicles with a diameter of approximately 30 to 150 nm and a density of 1.13 to 1.21 g/ml, containing RNA, protein, miRNA, DNA fragments, and other components. It can exist in body fluids such as blood, saliva, urine, and breast milk. Almost all types of cells including tumor cells can produce and release exosomes. Studies have found that exosomes can not only serve as carriers for information transmission between cells but also protect the miRNA carried in them and slow their degradation. Studies have shown that the degradation rate of exosomal encapsulated miRNAs is significantly lower than that of miRNAs without exosomes (30). A variety of exosome miRNAs mediate bioinformation transmission between the tumor and the tumor microenvironment and participate in PLC cell proliferation (31, 32), metastasis (33–35), immune escape, and other processes (36). Exosome miRNAs play an important role in the diagnosis, prognosis (37–39), and treatment (40–44) of PLC. Therefore, we hope to further clarify the pathogenesis of liver cancer by analyzing miRNA in rat plasma exosomes.

miRNA target prediction and function analysis can help reveal the possible function and mechanism of ATX prescription for treating abnormal miRNA in PLC. Therefore, in this study, GO analysis and KEGG pathway analysis were performed on the identified differential miRNAs. The results showed that the target genes of the differentially expressed miRNAs in the ATXP group *vs*. the model group were mainly involved in cellular processes, metabolic processes, and biological regulation. Additionally, the majority of these proteins were found to be located within the nucleus, cytoplasm, and intracellular organelles. Their primary functions were identified as protein binding, peptide binding, amide binding, and enzyme binding. It indicated that these miRNAs regulated the genetic network in the treatment of PLC by ATXP. Furthermore, with regards to the regulation of signal pathways associated with each group, the miRNAs identified in the ATXP group might play a role in regulating several pathways, including but not limited to the PI3K-Akt, MAPK, Ras, Wnt, mTOR, FOXO, TNF, and VEGF signaling pathways. As these pathways were associated with inflammation, apoptosis, and angiogenesis, it was plausible that these miRNAs were found to be differentially expressed in the ATXP group may have an impact on the development of PLC by regulating the target genes involved in these pathways.

We preliminarily selected the differentially expressed miRNAs in the PLC model rats after the intervention of ATXP by using Illumina sequencing. The results suggested that ATX prescription regulated the expression of these 14 miRNAs in rat plasma exosomes to some extent (>1.5- or <0.67-fold change in expression, *p* < 0.05) (as shown in **Table 4**). Among the identified miRNAs, the ATXP group showed upregulation of miR-339-5p, miR-223-5p, miR-147, miR-223-3p, miR-431, miR-199a-3p, miR-98-3p, and miR-99a-5p, while downregulation was observed for miR-125b-1-3p, miR-134-5p, miR-206-3p, miR-9a-5p, miR-674-3p, and miR-133a-5p. Researches have shown that among these exosomal miRNAs reversed by ATXP, miR-431, miR-223-5p, miR-339-5p, miR-223-3p, miR-99a-5p, miR-199a-3p, and miR-147 could inhibit a variety of tumors (including liver cancer) through multiple pathways (45–51). For instance, miR-431 could inhibit the migration and invasion of PLC cells by inhibiting Zeb1-mediated epithelial–mesenchymal transition (EMT) (52). As an independent prognostic factor in patients with liver cancer, miR-339-5p was lowly expressed in liver cancer tissues, which was able to inhibit tumor invasion, and its expression level was positively correlated with the overall survival rate of patients (53). miR-223-3p could inhibit HCC cell proliferation and promote apoptosis by directly targeting NLRP3 (54). The expression of miR-99a-5p in HCC was decreased, and it might inhibit the invasion and migration of HCC cells by targeting IGF1R (55). Downregulation of miR-199a-3p was a common feature of HCC; its reduced expression contributed to the activation of the mTOR and C-Met pathways (56), and it inhibited the proliferation, migration, invasion, metastasis, and angiogenesis of HCC cells through mitochondria-related apoptotic pathways (57). Moreover, although there was no direct evidence that miR-206-3p was related to liver cancer, it had the ability to reduce neuropathic pain and delay the symptoms of patients (58), which was consistent with the effect of ATXP on improving the condition and relieving the pain of patients with liver cancer. miR-674-3p, miR-125b-1-3p, miR-98-3p, miR-134-5p, miR-9a-5p, and miR-133a in **Table 4** had not been reported in PLC and other tumors, which may provide direction for our subsequent research.

Among the above-mentioned miRNAs closely related to liver cancer, we selected miR-199a-3p, which was upregulated in the ATXP group vs. the model group and had the smallest differential expression, as the research object for follow-up experiments. miR-199a-3p had been identified in recent years as a promising diagnostic biomarker in a variety of malignancies, such as liver cancer, colorectal cancer (59), lung cancer (60), esophageal cancer (61), and ovarian cancer (62). The study found that miR-199a-3p was underexpressed in liver cancer, which was in line with our sequencing results. ATXP could inhibit the occurrence and development of liver cancer by reversing the upregulation of miR-199a-3p. The target genes of miR-199a-3p were obtained from the miRDB, miRWalk, and TargetScan databases, respectively, and the GeneCards database was used to screen targets related to liver cancer. The final intersection of the 12 targets was used as candidate genes. Binding sites between miR-199a-3p and MAP3K4 were found by the TargetScan database. MAP3K4 (Mitogen-Activated Protein Kinase Kinase Kinase 4), also known as MTK1, MEKK4, MAPKKK4, PRO0412, etc., is a component of the protein kinase signal transduction cascade. The MAP3K4–p38 MAPK signal cascade pathway plays an important role in tumor invasion and metastasis. It has been confirmed that the p38 MAPK signaling pathway is associated with EMT and liver cancer metastasis: p38 plays an vital role in regulating IL-6 production and MAPK plays a synergistic role with the transcription factor SNAIL to promote tumor invasion and metastasis (63). Subsequently, in order to verify the relationship between miR-199a-3p and MAP3K4, a double luciferase experiment was used to confirm the high binding effect between them. It can be inferred that the mechanism of ATXP improving liver function and pathological injury in DEN-induced hepatoma rats may be through the inhibition of MAP3K4 expression by the plasma exosome miR-199a-3p. Therefore, our study revealed a new understanding of the mechanism of action of plasma exosome miRNAs in liver cancer. More importantly, this cellular communication between plasma exosomes and liver cancer cells linking ATXP might provide new options for future prophylactic therapy and contribute to the development of personalized diagnosis and treatment of liver cancer.

TCM compounds have complex components and diverse targets, and a single TCM or multiple components of a single TCM can regulate both the same miRNA and multiple miRNAs. Although TCM compounds increase the difficulty of the study, they provide a better choice for the comprehensive study of the miRNA interaction network. Therefore, we can conduct not only miRNA intervention from multiple levels, such as TCM monomer and single TCM, but also even TCM compounds, so as to discover new drugs, new targets, and new ways to treat diseases. Given the feasibility of miRNA intervention technology as a disease treatment method, TCM compounds can be used as miRNA modulators, which not only expand the theoretical connotation and clinical application of traditional Chinese medicine but also provide a new way for disease treatment and new drug research and development. In the future, we will explore the expression levels of mRNA and protein from the perspective of proteomics, making full use of the differences and complementarity of transcriptome and proteome studies. By conducting a comprehensive measurement of gene expression levels, we aim to fully understand the expression and regulation of each step of gene expression, and explore new findings that could not be found by conventional single omics approaches.

Although this study for the first time found that ATXP interferes with differentially expressed miRNAs in plasma exosomes of hepatoma model rats and simply verified the relationship between one of the miRNAs and its target genes, the specific regulatory mechanism has not been further studied. The causal relationship between microRNA expression and biological function is still very complex and mysterious, and the subsequent in-depth study and verification of a single miRNA is our future development direction. In addition to the previously reported miRNAs, we also identified several new PLC-related miRNAs. However, the targets of these miRNAs and the occurrence and development of PLC, as well as how ATXP affects the expression of target proteins in PLC rats, remain unclear. In the future, we will explore and study the pathogenesis of PLC affected by these miRNAs at the molecular level and the possible mechanism of multilevel and multitarget treatment of PLC by traditional Chinese medicine. With their help, we hope to provide new insights into the prevention and treatment of PLC.

## 5 Conclusion

In summary, this study provided preliminary evidence that ATXP might have downregulate the expression of MAP3K4 through the plasma exosome miR-199a-3p to further improve liver function and pathological injury in rats with liver cancer, which ultimately delayed the progression of liver cancer. Nevertheless, the specific mechanism of how ATXP mediates MAP3K4 through miR-199a-3p still requires more in-depth research for a comprehensive understanding.

## Data availability statement

The datasets presented in this study can be found in online repositories. The names of the repository/repositories and accession number(s) can be found in the article/supplementary material.

## Ethics statement

The animal study was reviewed and approved by the Animal Ethics Committee of Guangxi University of Chinese Medicine (Ethical approval code: DW20201226-91, Guangxi, China).

## Author contributions

LX and JC: investigation, formal analysis, writing—original draft preparation, and writing—reviewing and editing. ZL, XW, and YS: resources, methodology, and validation. MX and JL: conceptualization, supervision, project administration, and funding acquisition. All authors contributed to the article and approved the submitted version.
